# Combining free muscle flap lower limb reconstruction with targeted muscle reinnervation: A novel strategy to prevent neuropathic pain and promote sensory restoration

**DOI:** 10.1016/j.jpra.2026.09.026

**Published:** 2026-09-23

**Authors:** Camille Blaaker, Mirte Langeveld, Antonius J.M. Luijsterburg, J. Michiel Zuidam, Caroline A. Hundepool

**Affiliations:** a Department of Plastic, Reconstructive and Hand Surgery, Erasmus MC, Rotterdam, The Netherlands

**Keywords:** Targeted muscle reinnervation, Free muscle flap, Neuropathic pain, Limb salvage, Sensory reinnervation, IV

## Abstract

**Background:**

Severe lower extremity trauma with extensive tissue loss is often reconstructed using free muscle flaps. However, it involves vascular anastomosis but no nerve coaptation at the recipient site. Consequently, transected nerves have no opportunity to restore continuity, which can lead to neuroma formation and chronic neuropathic pain. Preventative targeted muscle reinnervation has been shown to facilitate reinnervation and reduce neuroma-related pain. This case series introduces a novel approach integrating targeted muscle reinnervation with free muscle flap reconstruction.

**Methods:**

A prospective case series was conducted at the Erasmus University Medical Center between April 2024 and June 2025. Surgical details and patient characteristics where documented, with patient- and clinician-reported outcomes assessed at 6 and 12 months.

**Results:**

Three patients with large soft tissue defects after traumatic injury of their lower extremities underwent reconstruction with a vascularized free muscle flap combined with targeted muscle reinnervation. At one year, no neuropathic pain was observed and two patients experienced partial return of deep pressure sensation. Throughout follow-up, patients reported only mild to moderate reductions in lower extremity function, stable or improved perceived quality of life and were highly satisfied with the procedure.

**Conclusion:**

Targeted muscle reinnervation combined with a free muscle flap is a technically feasible and functionally promising strategy for nerve management in soft tissue reconstruction, potentially enabling effective partial sensory restoration and potentially preventing neuroma-related pain. These findings support further investigation for broader clinical use in traumatic and elective reconstruction.

## Introduction

Severe traumatic injuries to the lower extremities pose a complex surgical challenge due to the often extensive soft tissue and bony defects.^1^^,^^2^ A commonly utilized and effective technique when dealing with large and complex soft tissue defects is reconstruction with a vascularized free muscle flap, to provide coverage for vital structures and orthopedic hardware, obliterate dead space, improve wound and bone healing, and reduce the risk of infection.^3^, ^4^, ^5^

During inset of the free muscle flap, vascular anastomosis between the flap and the recipient site is performed, but nerve coaptation is not. Consequently, injured nerves in the limb have no opportunity to restore continuity, which can lead to neuroma formation and chronic neuropathic pain.^6^, ^7^, ^8^, ^9^ This is a common consequence following traumatic lower limb surgery, with approximately 30% of patients experiencing neuropathic pain.^10^ The management of this chronic pain is particularly challenging, as conventional analgesics provide inadequate relief for approximately half of patients.^11^ Therefore, neuropathic pain often significantly impacts a patients' physical and physiological well-being, daily functioning and postoperative recovery.^8^^,^^10^^,^^12^^,^^13^

To combine soft tissue reconstruction with the provision of a physiologic target for transected nerve endings and aid in preventing neuropathic pain development, we propose a novel strategy in which the nerves of the free muscle flap are coapted to the injured nerves of the limb using targeted muscle reinnervation (TMR).^14^, ^15^, ^16^ In TMR, a transected peripheral nerve is redirected to a motor nerve branch supplying a portion of muscle, thereby providing a physiological target for axonal regrowth to prevent neuroma formation and subsequent neuropathic pain, with the additional potential for sensory reinnervation of both the muscle and skin.^17^, ^18^, ^19^, ^20^, ^21^ In amputation surgery, TMR has already been proven effective in reducing postoperative neuropathic pain and promoting motor sensory reinnervation.^17^, ^18^, ^19^, ^20^ Given the widespread use of free muscle flaps for reconstruction after traumatic injury, the question arises whether this technique can be effectively combined with TMR. Therefore, in this case series, we present three cases illustrating this novel reconstructive approach that integrates targeted muscle reinnervation with free muscle flap reconstruction.

## Methods

### Study design and setting

Between April 2024 and June 2025, we conducted a prospective case series at Erasmus University Medical Center, involving patients who underwent lower extremity soft tissue reconstruction with a vascularized free muscle flap in combination with TMR to a nerve target within the flap. All patients had a clinical indication for free muscle flap reconstruction following traumatic soft-tissue loss. TMR was discussed preoperatively as an adjunctive procedure to potentially reduce neuroma formation and neuropathic pain, and all patients consented. No eligible patients declined TMR.

Approval for this study was obtained from the medical ethics review board of the Erasmus Medical Center. The study was conducted and reported in accordance with the STROBE guidelines^22^ (Document, Supplementary Digital Content 1).

### Outcomes

Outcome measures were collected 6- and 12-months post-surgery to assess the presence of neuropathic pain, nociceptive pain, sensory recovery of the flap, functional status of the limb, and quality of life of the patients.

Neuropathic pain and nociceptive pain were evaluated using the 11-point Numerical Rating Scale (NRS).^23^ An NRS-score of ≥4 was considered indicative of significant pain. To distinguish neuropathic from nociceptive pain, patients completed the Dutch short-form Douleur Neuropathique 4 (s-DN4) questionnaire. The s-DN4 consists of seven items, with a score of ≥4 suggesting the presence of neuropathic pain.^24^ Neuropathic pain was consequently defined as a combination of an NRS-score ≥4 in combination with an s-DN4 score ≥4. Consistent with previous studies highlighting the clinical relevance of pain stratification in amputees based on its impact on quality of life (QoL).^8^^,^^25^ QoL was assessed using the Dutch EQ-5D-5L, measuring five dimensions of health: mobility, self-care, usual activities, pain/discomfort, and anxiety/depression. Responses yield a single index score ranging from 0 (death) to 1 (full health).^26^^,^^27^ Disability and limb function were assessed using the Dutch Lower Extremity Functional Scale (LEFS), which comprises 20 questions about daily activities, each rated on a 5-point ordinal scale of difficulty.^28^ Treatment satisfaction was evaluated using the Dutch ICHOM Satisfaction with Treatment Result (SWTR) questionnaire.^29^ The questionnaires and their corresponding scoring systems are provided in Supplementary Digital Content 2 (Questionnaires and Scoring Systems).

Lastly, sensory reinnervation of the free flap was assessed clinically with a combination of two tests conducted at 6- and 12-months post-surgery, the PinPrick stimulator and the Computerized Pressure Pain (CPP) Algometer.^30^, ^31^, ^32^, ^33^ The PinPrick stimulator was used to assess superficial cutaneous sensation. The CPP Algometer was used to evaluate somatosensory function of the underlying soft tissues by assessing deep pressure sensation. The patient was instructed to indicate when the sensation was first perceived, with the pressure threshold recorded in kilopascals (kPa). Clinical assessment using the CPP Algometer was conducted only in patients weightbearing on the free muscle flap, as deep pressure sensation is particularly relevant in this group and may serve as an important additional functional outcome beyond pain prevention.

Patient characteristics, surgical details and the presence of adverse events were recorded from the medical records.

## Results

Three patients who underwent TMR combined with a free muscle flap for lower extremity soft tissue reconstruction were included in this case series. A case description for each is presented below (Table 1).

Case 1:

**Table 1:** Clinical and surgical characteristics.

**Cases**	**Gender**	**Age**	**Reconstruction site**	**Soft tissue defect**	**Recipient vessels**	**Recipient nerve(s)**	**Free muscle flap**	**Flap vessels**	**Flap nerve**
1	M	45	Forefoot	Lisfranc amputation	Anterior tibial artery, Vena comitans	Superficial and deep peroneal nerve	Ipsilateral gracilis muscle	Medial circumflex femoral artery, Vena comitans	Obturator nerve
2	M	62	Forefoot	Lisfranc and cuboid amputation	Posterior tibial artery, Vena comitans	Tibial nerve	Contralateral latissimus dorsi muscle	Thoracodorsal artery, Vena comitans	Thoracodorsal nerve
3	F	8	Lower leg	Gustilo grade 3 open cruris fracture	Posterior tibial artery, Vena comitans	Saphenous nerve	Ipsilateral gracilis muscle	Medial circumflex femoral artery, Vena comitans	Obturator nerve

A 45-year-old male patient with type 2 diabetes mellitus sustained a severe crush injury to the left foot following entrapment between two forklifts, resulting in multiple metatarsal fractures, treated with a combination of plate-, trans-, and intramedullary fixation techniques. Postoperative healing was complicated by extensive oedema and suspected soft tissue necrosis one-month post-surgery, necessitating a forefoot amputation at the Lisfranc level combined with soft tissue reconstruction. An ipsilateral gracilis free muscle flap was selected based on the size and location of the forefoot defect, the availability of the anterior tibial artery, and the favorable orientation of the flap and its motor nerve. As both the superficial and deep peroneal nerves were transected within the zone of injury, these nerves were selected for TMR. The gracilis muscle flap was harvested from the ipsilateral leg and included a branch of the medial circumflex femoral artery, vena comitans and motor branch of the obturator nerve. In the lower leg, the anterior tibial artery and vena comitans were identified, as well as the transected superficial and deep peroneal nerves. The flap was inset with its distal portion anchored near the heel. Anastomosis of the anterior tibial artery, the medial circumflex femoral artery and venae comitans were performed with Ethilon 8.0, achieving successful revascularization within 42 minutes. TMR was subsequently performed of the superficial and deep peroneal nerves to motor branches of the obturator nerve using Ethilon 8.0 and Tisseel adhesive for additional stability. To cover the gracilis flap, a split-thickness skin graft (SSG) was harvested from the medial left thigh, meshed, and secured. A posterior lower-leg splint was subsequently applied to prevent pressure on the flap.

The healing process of the foot and gracilis flap progressed favorably, demonstrating stable integration and vitality. By approximately 3 months postoperatively, the patient achieved the ability to bear weight and ambulate on the stump (Fig. 1). The postoperative course at the donor site was complicated by a wound infection due to an infected hematoma, which resolved after surgical evacuation.

**Fig. 1:**
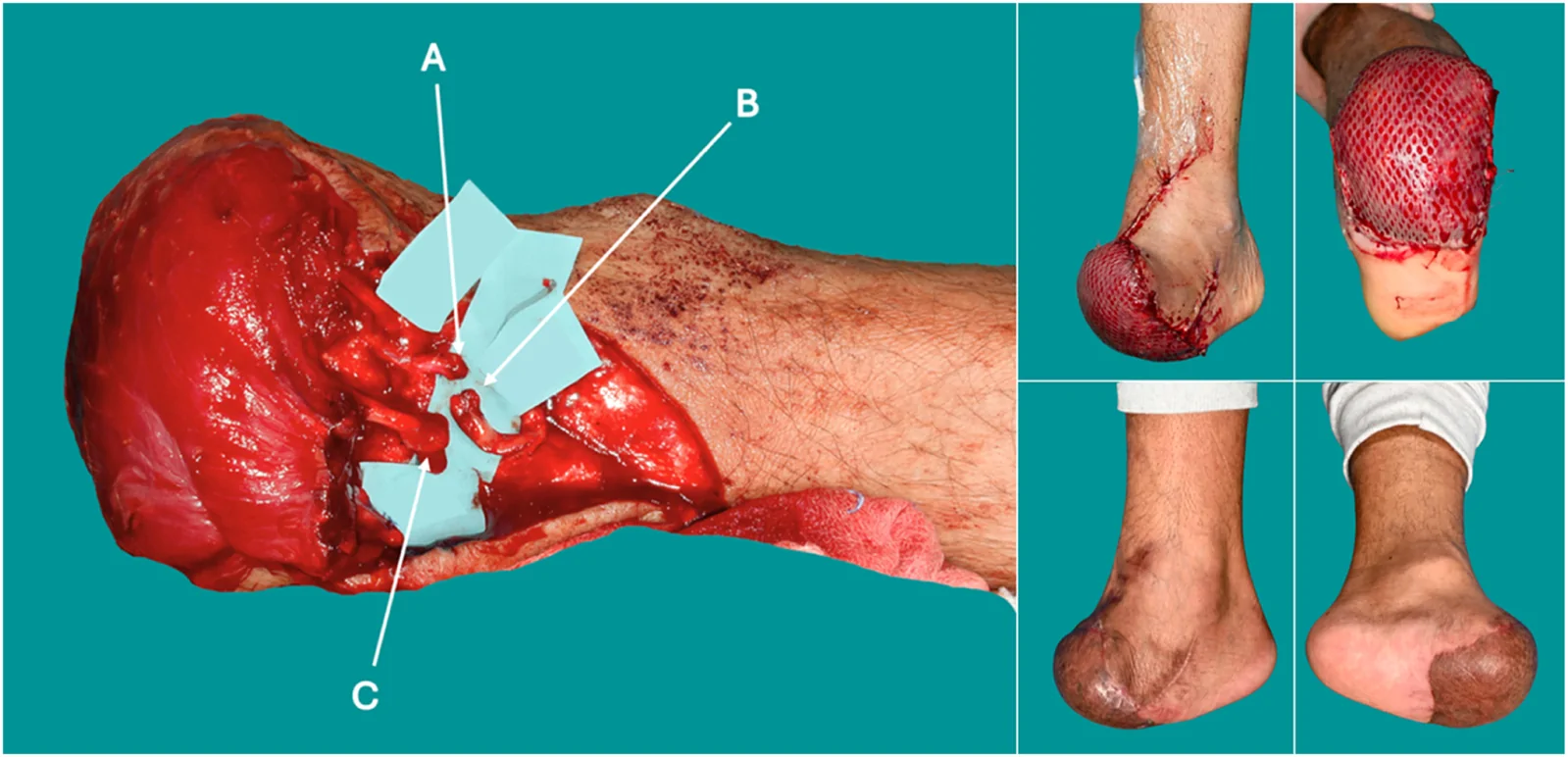
**Case 1.***(Left)* Intraoperative view of the free gracilis muscle flap with identification of the deep (A) and superficial (B) peroneal nerves, and the obturator nerve (C). (*Above, right*) Postoperative appearance of the amputated limb reconstructed with a free gracilis muscle flap and split-thickness skin graft (SSG). (*Below, right*) Healed residual limb following reconstruction.

At both 6- and 12-months post-treatment, the patient reported only short-lasting phantom limb sensations (PLS) in the amputated forefoot, occurring a few times per month, described as electric shocks and prickling, accompanied by a mild pain (NRS = 2), that did not meet the criteria for neuropathic pain (s-DN4 < 4). The patient's health-related QoL, as measured by the EQ-5D-5L, reflected a good but suboptimal health state, with an index value of 0.813 at 6 months and 0.743 at 12 months. Lower extremity function, as assessed with the LEFS, showed a slight increase from 6 months to 12 months postoperatively from 51/80 (63.8%) to 54/80 (67.5%), indicating moderate functional impairment at both time points. Patient satisfaction, measured by the ICHOM SWTR, remained high at both follow-up points.

Sensory testing using a PinPrick stimulator revealed no sensation on the SSG at either time point (no response to maximum 512 mN), while intact sensation was present on adjacent foot skin (response at minimum 8 mN). Pressure pain threshold testing with the CPP Algometer showed no deep sensation in the stump at 6 months. However, by 12 months, deep sensitivity was detected during clinical assessment dorsolateral and plantarly, with the patient reported perceiving pressure at 836.9 kPa dorsolateral and 407.7 kPa plantarly, though no sensation was reported medially (Table 2).

Case 2:

**Table 2:** Clinical and patient-reported outcomes at 12-months.

**Cases**	**NRS Nociceptive pain**	**Neuropathic limb pain**	**Phantom limb pain**	**Return of sensibility**	**Lower Extremity Functional Scale**	**EQ-5D-5L** **Quality of life**
	***(0-10)***	***(yes/no)***	***(yes/no)***		***(/80)***	***(0-1)***
						
1	2	No	No	Deep sensation	54/80 (67.5%)	0.743
2	2	No	No	Deep sensation	63/80 (78.8%)	0.620

Abbreviations: NRS Numerical Rating Scale

A 62-year-old man with a history of alcohol and tobacco use, hypertension, and obesity presented to the emergency department with severe crush injuries to the left lower leg and foot after a collision with a truck while cycling. The extensive soft tissue injuries and necrosis necessitated a combined Lisfranc and cuboid amputation with removal of a large portion of the remaining skin over the calcaneus. The procedure was combined with soft tissue reconstruction using a vascularized free latissimus dorsi muscle flap. The latissimus dorsi muscle flap including the thoracodorsal nerve, artery and vena comitans were harvested from the contralateral side. Additionally, the posterior tibial artery and vena comitans were exposed in the tarsal tunnel. The free muscle flap was chosen based on the extent of the forefoot defect, the availability of the posterior tibial artery, and the favorable orientation of the flap and its motor nerve. The tibial nerve was injured and anatomically well accessible at the recipient site, it was therefore dissected, preserving the calcaneal branch, and transected just distal to the tarsal tunnel for TMR. The latissimus dorsi muscle flap was inset, and microvascular anastomoses were performed between the posterior tibial artery, thoracodorsal artery and venae comitans. TMR was then carried out by connecting the tibial nerve to the thoracodorsal nerve using Ethilon 8.0 and Tisseel adhesive. SSGs were harvested and applied to the latissimus dorsi flap. Finally, an external fixator was applied for stabilization and to offload the reconstructed area.

The foot and latissimus dorsi muscle demonstrated good viability postoperatively with stable integration and gradual thinning over time (Fig. 2). Two months after the forefoot reconstruction, the patient started bearing weight on the leg and flap while wearing a cast splint. Throughout the 6- and 12-month follow-up period, the patient reported no clinically significant pain and did not meet the predefined criteria for neuropathic pain. At 6 months follow-up the patient reported occasionally experiencing brief episodes of tingling and electric-shock-like sensations (NRS = 2, s-DN4 < 4) in the residual limb, occurring once every few weeks, which had resolved at 12-months follow-up. Health-related quality of life showed a modest improvement over time. The EQ-5D-5L index increased from 0.511 at 6 months to 0.620 at 12 months, with better perceived functioning in self-care and daily activities after one year. Functional ability, as measured by the LEFS, improved from 40/80 (50%) at 6 months to 63/80 (78.8%) at 12 months, indicating an increase from moderate to mild functional impairment. Satisfaction with the treatment outcome, measured with the ICHOM SWTR, remained consistently high at 6 and 12 months.

**Fig. 2:**
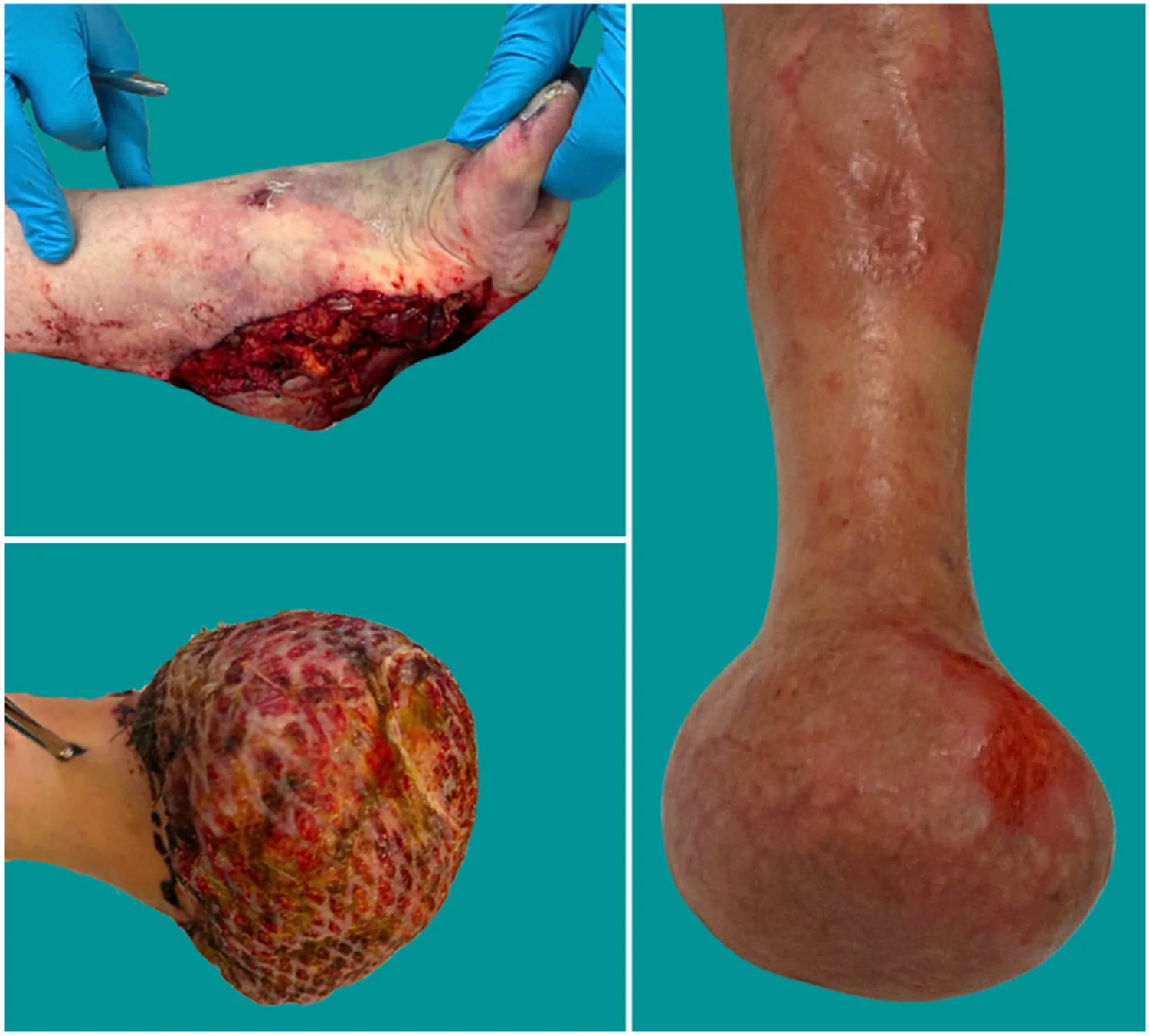
**Case 2.** (*Above, left*) Extensive soft tissue damage of the foot following severe trauma. (*Below, left*) Reconstruction of the amputated limb using a free latissimus dorsi flap and split-thickness skin graft (SSG). (*Right*) Well-healed residual limb following reconstructive surgery.

Sensory innervation was tested at 12 months. PinPrick stimulation over the skin graft indicated no superficial cutaneous sensation at the maximum applied force (512 mN). Pressure pain threshold testing using a computerized algometer demonstrated deep sensory perception medially at 684 kPa and laterally at 465.5 kPa, with no response at dorsal, plantar, or axial sites of the stump (Table 2).

Case 3:

An 8-year-old girl sustained severe injuries in a road traffic accident. On presentation, the patient exhibited multiple traumatic injuries, including a Gustilo grade 3 open cruris fracture of her left leg. Initial surgical management included external fixation of the left cruris fracture. Four days post-trauma a second surgical procedure was performed to facilitate further reconstruction and stabilization of the injuries. The left lower leg presented with a significant soft tissue defect measuring 20 × 6 cm, exposing the dorsum of the foot and injury to the saphenous nerve. Based on the defect location and available recipient vessels, a gracilis muscle flap was harvested from the ipsilateral leg to reconstruct the soft tissue defect. The free muscle flap included a branch of the medial circumflex femoral artery, vena comitans and a branch of the obturator nerve. The flap was inset and microvascular anastomoses between the medial circumflex femoral artery, posterior tibial artery and venae comitans were performed. The traumatically injured saphenous nerve was coapted to the obturator nerve within the flap using Ethilon 8.0 sutures and Tisseel adhesive for stabilization. This was as a preventive TMR procedure, providing a physiological target for axonal regeneration and potentially reducing neuroma-related pain.^10^ An SSG, harvested from the left inner thigh and meshed 1:1.5, was applied to further cover the defect. An external fixator was applied to prevent foot drop and offload the gracilis muscle flap. Recovery was complicated by Pseudomonas colonization leading to localized necrosis and partial SSG detachment, for which debridement’s were performed. The flap remained viable, and after 3 months healing was complete (Fig. 3).

**Fig. 3:**
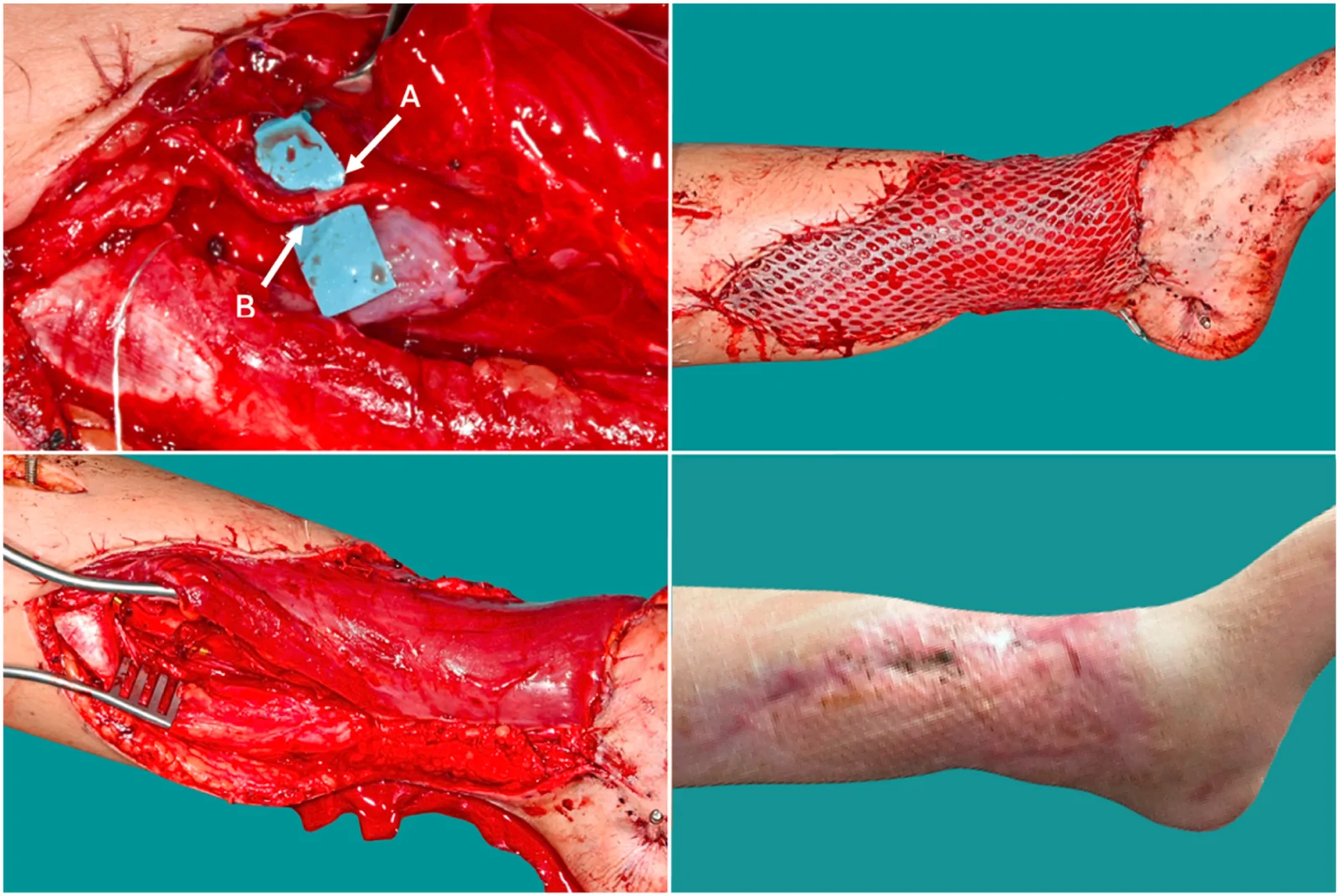
**Case 3.***(Above, left)* Targeted muscle reinnervation (TMR) of the saphenous (A) and obturator (B) nerves. *(Below, left)* Intraoperative inset of a free gracilis muscle flap for soft tissue defect coverage. *(Above, right)* Postoperative appearance following reconstruction with a free gracilis muscle flap and split-thickness skin graft (SSG). *(Below, right)* Fully healed reconstruction with stable SSG integration.

Neuropathic pain in the left leg and foot was reported around one-month post-surgery, prompting initiation of gabapentin and later amitriptyline. As wound healing progressed and no signs of allodynia or hyperalgesia were observed, neuropathic pain medication was gradually tapered and fully discontinued within 4 months. After 1 year, no neuropathic pain was present. Other secondary outcomes were not collected due to the age of the patient.

## Discussion

This case series demonstrates that TMR can be successfully performed within a free muscle flap following extensive traumatic injuries. This novel approach enables reinnervation, as evidenced by partial return of deep pressure sensation within the free muscle flaps. Furthermore, the achieved reinnervation may prevent neuroma formation, as no signs of neuropathic pain were observed one year postoperatively. Furthermore, patients exhibited stable tissue integration, preserved function, adequate quality of life, and high levels of satisfaction. Despite postoperative complications in two patients requiring additional surgical intervention, successful soft tissue healing was achieved within two to three months.

TMR was initially introduced by Kuiken et al.^34^ to enhance myoelectric prosthesis control for amputees, by reinnervating denervated muscle with residual peripheral nerves to generate additional, intuitive EMG control signals. In these patients, TMR demonstrated an improvement in neuroma-related pain. Souza et al.^18^ subsequently demonstrated that TMR can also be performed directly at the time of amputation to prevent neuroma formation and consequent neuropathic pain in amputees, becoming a key advancement in nerve pain prevention.^17^, ^18^, ^19^, ^20^ However, the combination of TMR with amputation and subsequent soft tissue reconstruction is still largely unexplored. Only two cases of TMR combined with free muscle flaps have been reported in the current literature, which are primarily aimed at optimizing myoelectric prosthesis control. Notably, neither case, observed neuroma formation or subsequent nerve pain after one year, findings that are consistent with our own results.^15^^,^^16^ In our study one patient reported experiencing suspected neuropathic pain, but this resolved within four months. The second patient continued to report occasional episodes of non-disabling phantom limb symptoms at one year, consistent with previous findings that this may persist following TMR.^35^^,^^36^ Our findings further support that preventive TMR can be safely performed even in lower extremity amputation with complex reconstructions involving free muscle flaps, leading to successful reinnervation and potentially preventing neuroma formation. Given the debilitating nature of neuropathic pain, its negative impact on recovery and the limited efficacy of conventional analgesia, this is of particular clinical relevance for patients undergoing free muscle flap reconstruction, not only after traumatic injury, but also in the context of elective amputations and oncologic limb salvage procedures.^8^^,^^10^^,^^12^^,^^13^

Further evidence of successful reinnervation was observed through partial recovery of deep sensation within the muscle flap in two patients. Deep afferent input through the muscle flap’s mechanoreceptors, is important because it supports proprioception and load feedback, which improves limb positioning, balance, and weight transfer during standing or movement, and may reduce pressure injuries and falls.^37^, ^38^, ^39^ Recent experimental work by Festin et al.^21^ provides important physiological insight into this process. In their animal model, sensory axons originating from a redirected mixed nerve traversed the reinnervated muscle and extended into the overlying skin graft through the musculocutaneous junction, reestablishing both cutaneous and proprioceptive feedback pathways. This mechanism likely underlies the observed restoration of deep sensory feedback; however, proprioceptive reinnervation remains a largely overlooked aspect in both clinical and experimental research. As most studies to date have focused primarily on cutaneous reinnervation.^40^ Although sensation in the overlying skin grafts remained absent, cutaneous reinnervation might still to be able to occur over time, though we do not know this at this timepoint. Evidence on the use of targeted muscle reinnervation in free flaps remains scarce, and long term outcomes are not reported, leaving the time course of cutaneous reinnervation in this setting uncertain.^14^, ^15^, ^16^ Studies on free muscle flaps, both with and without sensory nerve coaptation, show that cutaneous sensibility can continue to improve up to 24 months after the procedure.^40^, ^41^, ^42^, ^43^, ^44^ Overall, these findings suggest that, when applied in free flap reconstructions with a skin graft, TMR has the potential not only to restore bidirectional sensory-motor integration but also to support ongoing sensory recovery while preventing neuroma formation.

The choice of nerves and free muscle flaps varied between patients. Our study included three cases with extensive soft tissue defects: two following Lisfranc-level forefoot amputations and one after severe lower leg crush injury. Although both amputation cases involved the same anatomical level, differences in laterality of the injury, peripheral nerve involvement, vascular access, flap orientation, and motor nerve position and availability within the flap determined the final reconstructive approach. In both Lisfranc-level forefoot amputations, flap and nerve selection were guided by the defect location and available recipient vessels. In both cases, only the involved major sensory nerves were targeted for TMR, as complete denervation was not feasible due to the complex branching pattern at this level. In Case 3, the damaged saphenous nerve was intentionally denervated as a preventive measure, given that neuropathic pain occurs in approximately 30% of patients after traumatic lower limb surgery.^10^ Thus, variation in nerve and flap selection reflected not only the zone of injury and defect size, but also the available recipient vessels, flap orientation, and motor nerve entry points, underscoring the need to tailor the reconstructive and TMR strategy to each individual case.

This study has several limitations. In one case, some of the outcome measures, such as assessments of cutaneous and deep sensation, could not be completed due to patient age. Additionally, all included cases involved free muscle flap reconstruction following traumatic injury. Whether similar outcomes can be achieved in oncologic populations, where chronic pain is often pre-existing due to tumor burden, chemoradiation, or previous salvage procedures, remains uncertain.^45^ Future studies should include larger, more heterogeneous cohorts and longer-term follow-up, with systematic evaluation across both traumatic and non-traumatic indications to assess further sensory recovery and determine the broader applicability and effectiveness of TMR combined with free muscle flaps.

## Conclusion

Integrating targeted muscle reinnervation (TMR) in a free muscle flap is a technically feasible and functionally promising strategy for nerve management in soft tissue reconstruction. This novel approach may enable effective reinnervation, as evidenced by partial recovery of deep pressure sensation within the flaps, and may help prevent neuroma formation, as no signs of neuropathic pain were observed one year postoperatively. While this study provides an important foundation for broader clinical application in both traumatic and elective reconstructive settings, larger prospective studies with longer follow-up are warranted to confirm and expand upon these results given the limited number of cases.

## Funding

None.

## Declaration of competing interest

None declared.
