# Supplementary material for: Combining free muscle flap lower limb reconstruction with targeted muscle reinnervation: A novel strategy to prevent neuropathic pain and promote sensory restoration
Source: JPRAS Open. 2026 Sep 23;52:543–50. doi: 10.1016/j.jpra.2026.09.026 (PMC13639968; doi:10.1016/j.jpra.2026.09.026)
Supplement: Supplementary file 1 [file mmc2.pdf]

<u>11-point Numerical Rating Scale (NRS):</u>

Residual limb pain and discomfort:

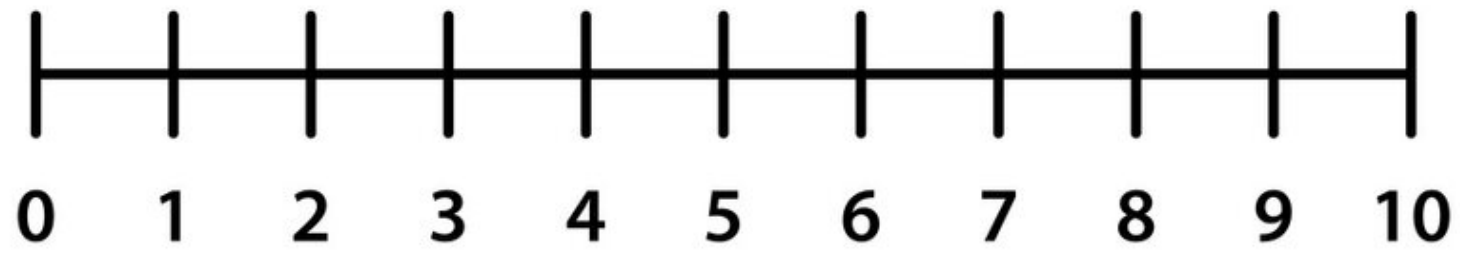

Phantom limb pain/sensation:

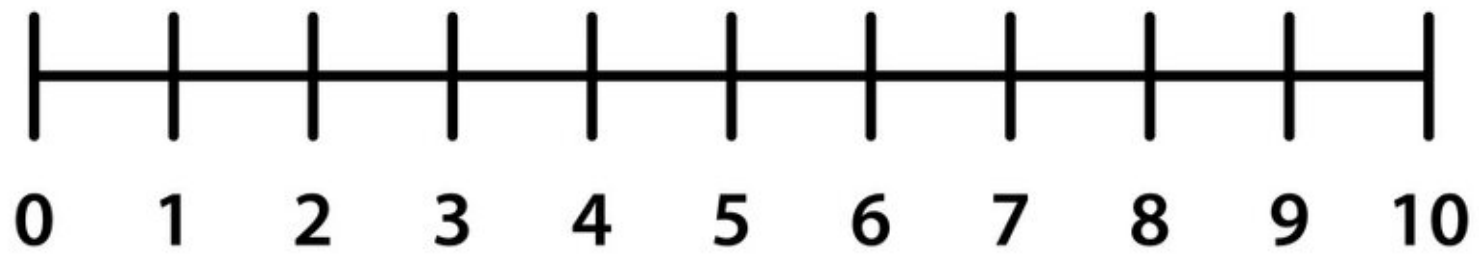

Dutch short-form Douleur Neuropathique 4 (s-DN4) questionnaire:

Heeft de pijn één of meer van de volgende kenmerken?	
Branderig	☐ JA ☐ NEE
Pijnlijk koude gevoel	☐ JA ☐ NEE
Elektrische schokken	☐ JA ☐ NEE
**Gaat de pijn gepaard met één of meer van de volgende symptomen in hetzelfde gebied?**	
Tintelingen	☐JA ☐ NEE
Prikken	☐ JA ☐ NEE
Doofheid	☐ JA ☐ NEE
Jeuk	☐ JA ☐ NEE
**Is er in het pijngebied t.o.v. een normaal aanvoelend gebied een verminderd gevoel bij**	
Aanraking	☐ JA ☐ NEE
Prikken (cocktailprikker)	☐ JA ☐ NEE
**Wordt de pijn verergerd door**	
Wrijven	☐ JA ☐ NEE

≥ 4 neuropathische pijn is waarschijnlijk:

Dutch EQ-5D-5L:

Door een vinkje te plaatsen in één vakje per groep hieronder, geef alstublieft aan welke uitspraken uw huidige gezondheidstoestand het beste beschrijven.

Mobiliteit

- ☐ Ik heb geen problemen met lopen
- ☐ Ik heb een beetje problemen met lopen
- ☐ Ik heb matige problemen met lopen
- ☐ Ik heb ernstige problemen met lopen
- ☐ Ik ben niet in staat om te lopen

Zelfzorg

- ☐ Ik heb geen problemen met mijzelf wassen of aankleden
- ☐ Ik heb een beetje problemen met mijzelf wassen of aankleden
- ☐ Ik heb matige problemen met mijzelf wassen of aankleden
- ☐ Ik heb ernstige problemen met mijzelf wassen of aankleden
- ☐ Ik ben niet in staat mijzelf te wassen of aan te kleden

Dagelijkse activiteiten (*Bijvoorbeeld werk, studie, huishouden, gezins- en vrijetijdsactiviteiten*)

- ☐ Ik heb geen problemen met mijn dagelijkse activiteiten
- ☐ Ik heb een beetje problemen met mijn dagelijkse activiteiten
- ☐ Ik heb matige problemen met mijn dagelijkse activiteiten
- ☐ Ik heb ernstige problemen met mijn dagelijkse activiteiten
- ☐ Ik ben niet in staat mijn dagelijkse activiteiten uit te voeren

Pijn/klachten

- ☐ Ik heb geen pijn of ongemak
- ☐ Ik heb een beetje pijn of ongemak
- ☐ Ik heb matige pijn of ongemak
- ☐ Ik heb ernstige pijn of ongemak
- ☐ Ik heb extreme pijn of ongemak

Stemming

- ☐ Ik ben niet angstig of somber
- ☐ Ik ben een beetje angstig of somber
- ☐ Ik ben matig angstig of somber
- ☐ Ik ben erg angstig of somber
- ☐ Ik ben extreem angstig of somber

We willen weten hoe goed of slecht uw gezondheid is. Zet een kruisje op de plek die het best overeenkomt met hoe uw gezondheid vandaag is.

Schrijf nu het nummer wat u heeft aangekruist hier: ....

Een score van **0** betekent de slechts mogelijke gezondheid die u zich kunt voorstellen

Een score van **100** betekent de best mogelijke gezondheid die u zich kunt voorstellen.

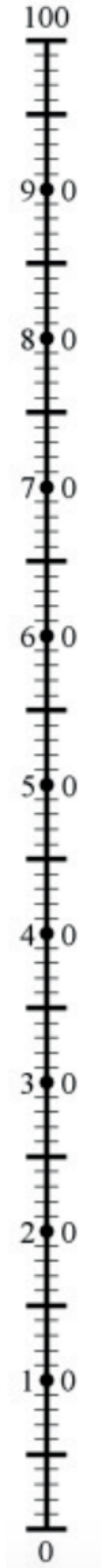

The Dutch Lower Extremity Functional Scale (LEFS):

LOWER EXTREMITY FUNCTIONAL SCALE (LEFS)

Datum: Geboortedatum:

Naam:

TOELICHTING: Deze lijst vraagt naar uw mening over uw beenfunctie. Uw antwoorden geven ons een beeld van uw klachten in de afgelopen week en hoe u in staat bent om alledaagse activiteiten uit te voeren.
Beantwoorden van een vraag doet u door het aankruisen van een vakje met het volgens u meest juiste antwoord (één vakje per vraag). Als u niet zeker weet hoe u een vraag moet beantwoorden, geeft u dan het antwoord dat volgens u het meest op uw situatie van toepassing is.

Controleer na het invullen van de lijst a.u.b. of alle vragen beantwoord zijn.

Bezigheden	Onmogelijk, heel veel moeite	Veel moeite	Moeite	Weinig moeite	Geen moeite
1. Een onderdeel van uw gebruikelijke werk, huishouden of schoolactiviteiten	0	1	2	3	4
2. Uw gebruikelijke hobby's, vrijetijdsbesteding of sportactiviteiten	0	1	2	3	4
3. In of uit bad stappen	0	1	2	3	4
4. Binnenshuis stappen	0	1	2	3	4
5. Uw schoenen of sokken aantrekken	0	1	2	3	4
6. Hurken	0	1	2	3	4
7. Iets van de grond optillen, zoals een tas met boodschappen	0	1	2	3	4
8. Lichte taken uitvoeren in en om uw huis	0	1	2	3	4
9. Zware taken uitvoeren in en om uw huis	0	1	2	3	4
10. In of uit een auto stappen	0	1	2	3	4
11. 250 meter stappen	0	1	2	3	4
12. Anderhalve kilometer stappen	0	1	2	3	4
13. Een trap op- of afgaan (ongeveer 10 treden)	0	1	2	3	4
14. Eén uur staan	0	1	2	3	4
15. Eén uur zitten	0	1	2	3	4
16. Lopen op een vlakke ondergrond	0	1	2	3	4
17. Lopen op een oneffen ondergrond	0	1	2	3	4
18. Tijdens het lopen scherpe bochten maken	0	1	2	3	4
19. Huppelen op het aangedane been	0	1	2	3	4
20. Omdraaien in bed	0	1	2	3	4

Dutch ICHOM Satisfaction with Treatment Result (SWTR) questionnaire:

1. **Hoe tevreden bent u met het resultaat van uw behandeling tot nu toe?**
   Kies a.u.b. één van onderstaande opties:

Uiterst ontevreden	Erg ontevreden	Enigzins ontevreden	Niet tevreden of ontevreden	Enigzins tevreden	Erg tevreden	Uiterst tevreden

Door een vinkje te plaatsen in één vakje per groep hieronder, geef alstublieft aan welke optie voor u van toepassing is.

2. **Zou u bij gelijke omstandigheden de behandeling opnieuw ondergaan?**
   ☐ Ja
   ☐ Nee

3. **Zou u deze behandeling aanbevelen aan vrienden en familie?**
   ☐ Ja
   ☐ Nee
